# Restoring Adipose Tissue Homeostasis in Response to Aging: Initial Clinical Experience with Profhilo Structura^®^

**DOI:** 10.3390/gels9080614

**Published:** 2023-07-28

**Authors:** Daniel Cassuto, Clara Cigni, Gilberto Bellia, Chiara Schiraldi

**Affiliations:** 1 Private Practice, 20129 Milan, Italy; 2 Private Practice, Tel Aviv 6971017, Israel; 3 IBSA Farmaceutici Italia Srl, 26900 Lodi, Italy; 4 Department of Experimental Medicine, Biotechnology, Medical Histology and Molecular Biology, University of Campania Luigi Vanvitelli, 80138 Naples, Italy

**Keywords:** hyaluronic acid, hybrid cooperative complexes, adipose tissue restoration

## Abstract

The aim of the case series was to determine the efficacy of a new medical device developed for adipose tissue restoration in the face. The medical device used the patented NAHYCO^®^ Hybrid Technology to deliver 45 mg of high- (1400 ± 200 kDa) and 45 mg of low- (100 ± 20 kDa) molecular-weight hyaluronan, in 2 mL. Patients and methods: Twenty-two volunteers, aged 36–60 years. Two mL of Profhilo^®^ Structura was injected using a 25 G cannula for each hemiface, into superficial fat compartment along the line from the preauricular area to the mandibular angle. Two injections were performed, and Profhilo Structura’s effect on restoring adipose tissue was evaluated immediately after treatment, and over a 6-month follow-up. The studied medical device revealed a pseudoplastic behavior and consistency that allowed easy extrusion from a syringe. It showed a lower viscosity compared to dermal fillers, based on crosslinked HA. Clinically, the soft tissue thickness increased immediately after injection, and the clinical improvement persisted across a 6-month follow-up. The self-reported satisfaction with the treatment showed an amelioration in the midface of all the subjects enrolled, with no adverse effects. Profhilo^®^ Structura demonstrated a peculiar fat compartment integration, with a regenerating effect on adipose tissue senescence. The skin thickening and compaction effects were similar to those obtained using chemically crosslinked dermal fillers, while a natural look was preserved, and the use of crosslinking agents was avoided.

## 1. Introduction

Age-related morphological changes in the face, such as wrinkles, fat and bone resorption, and skin dehydration are consequences of soft tissue elasticity and volume loss. In particular, the facial fat compartments have been proven to play a pivotal role in the aging process in the face [1]. A clinical investigation into the distribution of the adipose compartments around the facial rhytides revealed that different arrangement patterns may evolve during aging [2,3,4,5,6]. For this reason, fat compartment restoration is becoming a growing approach toward facial rejuvenation. The current practice has focused on the optimization of fat-grafting injection techniques aimed at augmenting the soft-tissue volume, or facial sculpting via lipotransfer [7,8].

Nevertheless, fat-tissue grafting, or fat transfer with human adipose stem cells presents various potential drawbacks because of the unpredictable results, such as a loss of transplanted adipose tissue that cannot tolerate hypoxia, and is often reported as readily liquefying. The clinical use of lipotransfer is also dependent on the development of a less invasive and safer fat-harvesting procedure [9,10].

Nowadays, soft-tissue filler injections represent an excellent option for minimizing the invasiveness of rejuvenation techniques, and is becoming a cornerstone of the procedures for skin thickness restoration and facial sculpting. Hyaluronic acid (HA) intradermal formulations have gained massive popularity in aesthetic medicine, due to HA’s ability to improve skin texture, and to re-activate dermal functions, such as collagen and elastin biosynthesis, leading to rejuvenation [11,12,13,14,15,16].

Although crosslinked dermal fillers generate immediate results in tissue contouring, by ensuring optimal safety and tolerability profiles, they do not excel in encouraging the local production of matrix proteins, due to their low biological activities [17].

The Profhilo^®^ product line is distinct from the various HA fillers on the market because it is based on hybrid cooperative complexes of high- and low-molecular-weight hyaluronans, abbreviated as HCCs [14]. HCCs have been developed with the use of NAHYCO^®^ technology (patent No. WO 2012/0321151 A2). With this technology, it is possible to create stable L/H-HA cooperative hybrids, by submitting aqueous solutions containing high-molecular-weight (1100–1400 kDa) HA and low-molecular-weight (80–100 kDa) HA together to a suitably configured thermal cycle. After the thermal process, the newly created HCCs are stable over time, and mantain their rheological characteristics. In particular, a low viscosity and high flowability allow the gel to spread easily and homogenously during extrusion through the needle and, after the injection, in the dermal tissue. HCCs’ rheological characteristics allow, therefore, the use of solutions with a higher HA concentration, compared to the cross-linked HA fillers commonly available on the market [14]. HCCs have also been widely investigated in the skin remodeling process, with in vitro studies showing that HCC stimulated elastin and collagen expression and synthesis in human keratinocyte (HaCaT) and fibroblast (HDF) monocultures and cocultures, contributing to refining the cell morphology, and potentially improving the skin function, elasticity, and global tissue homeostasis. In addition, HCCs have exhibited a prolonged stability to enzymatic attack, in spite of the lack of chemical modification (e.g., cross-linking) [14]. Further research has recently showed that Profhilo^®^ enhances the adipogenic differentiation and viability of human ASCs (adipose stem cells) more than linear HA and cross-linked hyaluronan-based gels [18]. The most recent and innovative formulation of the patented NAHYCO^®^ Hybrid Technology, known as Profhilo Structura^®^, is still composed of HCCs, but differs from the other products in the Profhilo^®^ line by its cohesivity, and by containing the highest concentration of HA, consisting of 45 mg of low-molecular-weight (80–100 kDa) HA and 45 mg of high-molecular-weight (1100–1400 kDa) HA, in 2 mL.

Due to its properties, Profhilo Structura^®^ showed a durability comparable to cross-linked gels when injected into a murine model but, at the same time, it also displayed a better reversibility of the treatment, demonstrating a higher safety profile compared to chemical cross-linked HA fillers [19].

Based on the HCC in vitro findings on ASCs [18], the present work aimed to evaluate the clinical efficacy of the new medical device in tissue renewal in the facial zones characterized by gravitational ptosis and volume deflation, by directing human ASCs toward the adipogenic phenotype.

The retrospective case series reported herein aimed to understand the specific action of the injection in the fat compartment toward the regeneration of the lower layer, as well as evaluating the product–tissue integration. To better investigate and predict the tissue distribution and integration of Profhilo Structura^®^ after injection within the fat compartments, the cohesivity was also determined. In addition, this study also aimed to evaluate the safety of the medical device, measured as the absence of product-related adverse reactions.

## 2. Results and Discussion

### 2.1. Cohesivity

**Figure 1:**
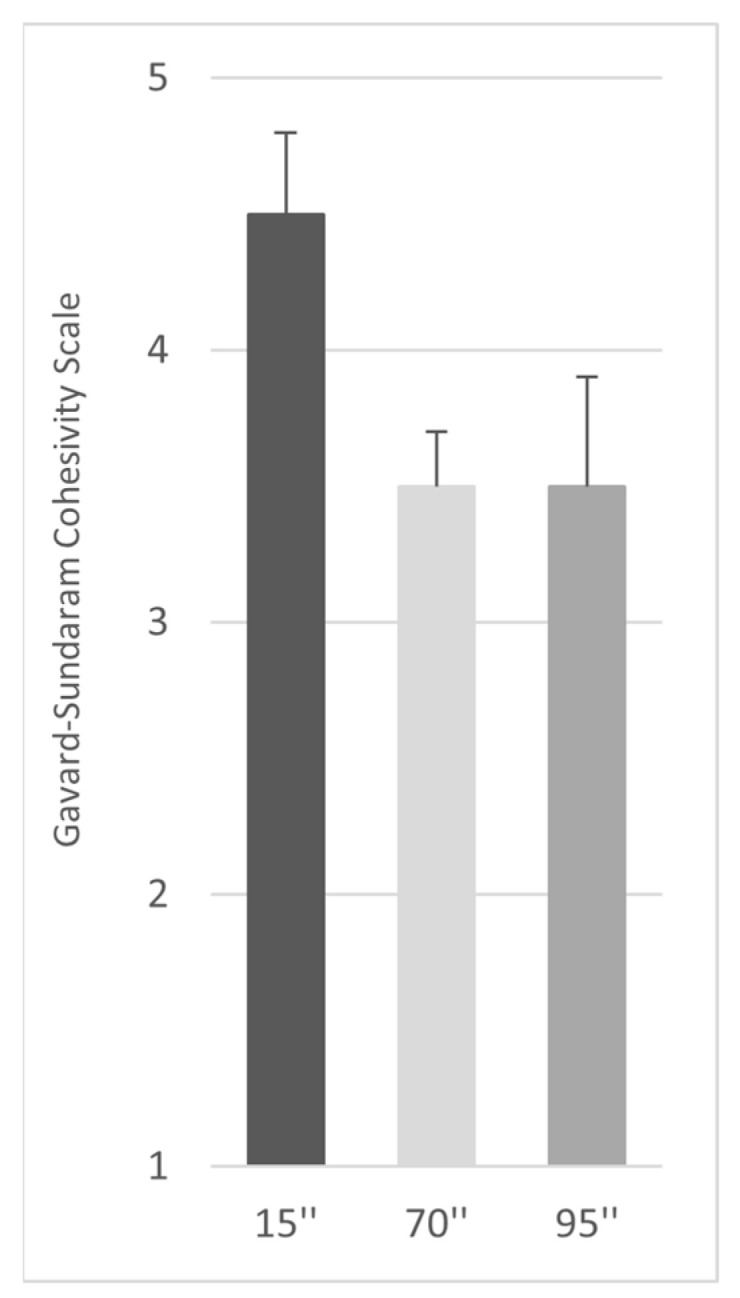
The cohesivity properties of Profhilo^®^ Structura. The panel reports a representative result of the tests performed at 15, 70, and 95 s.

Cohesivity is described as the force between particles of the same substance that acts to unite them [20,21]. In this context, it is a measurement of the property of the gel to not dissociate when subjected to force. The Sundaram–Gavard scale was used to evaluate the gel cohesivity at 15, 70, and 90″, and the cohesivity scores obtained are reported in Figure 1. Profhilo Structura^®^ showed a high cohesivity score, up to 4.5 at 15″, and up to 3.5 at 70 and 90″, indicating the ability of the gel to highly preserve its structure within the tissue, without altering its spreadability.

### 2.2. Clinical Evaluations

At the end of this retrospective case series, 22 subjects (20 females and 2 males) were enrolled with the following characteristics: the mean age was 53 years (range: 36–60), while the mean body mass index (BMI) was 21 (range: 18–28).

The evaluation of the treatment efficacy was performed using the US measurement of skin thickness, performed during the basal visit, and before the first treatment (T1), the second visit (T2), and the follow-up visits three (T3) and six months (T4) after the first treatment.

**Figure 2:**
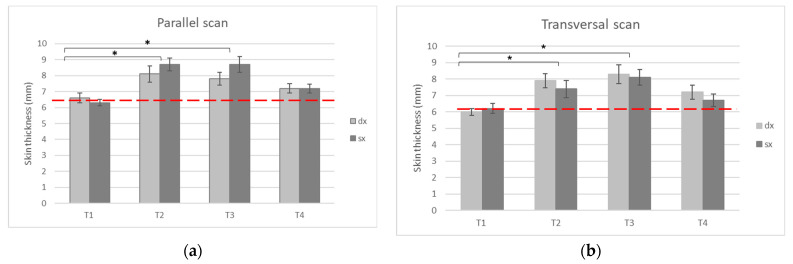
The US skin thickness determination (mm) using a parallel (**a**) or transversal (**b**) scan in both the right and left side. The measurements were taken before treatment (T1), 30 days after the first treatment and during the second treatment (T2), and three (T3) and six (T4) months after the beginning of the treatment. The red dashed lines indicate the baseline measurement media, and the statistically relevant differences are reported (* = *p* value < 0.05).

The skin thickness evaluations were performed on the preauricular area of both the left and right side of the face, using transversal and parallel scans. The measurements clearly showed a statistically significant increase in the skin thickness values (*p* values < 0.05) on both the US transversal and parallel scans, after treatment and, especially, at T2 and T3 (Figure 2).

Importantly, the positive effects of the injections were maintained, and were statistically significant (*p* value < 0.05) three months after the first treatment, and two months after the end of treatment. Although a decrease trend in the skin thickness values was visible at T4, and the data were not statistically significant at this time point, the effects of the treatment were also still observable six months after the beginning of the treatment (Figure 2).

**Figure 3:**
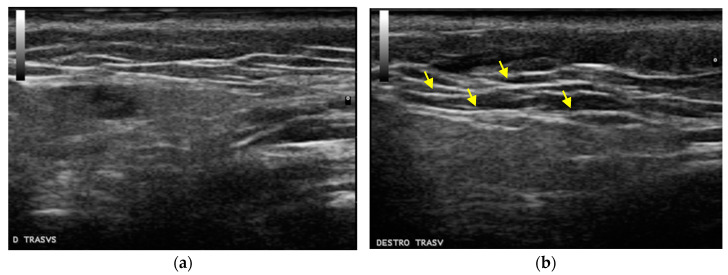
The US qualitative scan evaluation before treatment (**a**) and three months after the first treatment (**b**), with an increased thickness in the subcutaneous layer. The yellow arrows indicate the fat hyperechoic areas.

Moreover, the US scans were also evaluated qualitatively, in order to evaluate the product–tissue integration. Hyperechoic areas are classically associated with fat in the US analysis, due to the adipose tissue acoustic impedance relative to the surrounding tissue. In this regard, an increase in hyperechoic areas and, therefore, an increase in the thickness of the injected fat compartment, could still be observed 30 days after the first treatment (T2), when the second visit occurred (Figure 3).

**Figure 4:**
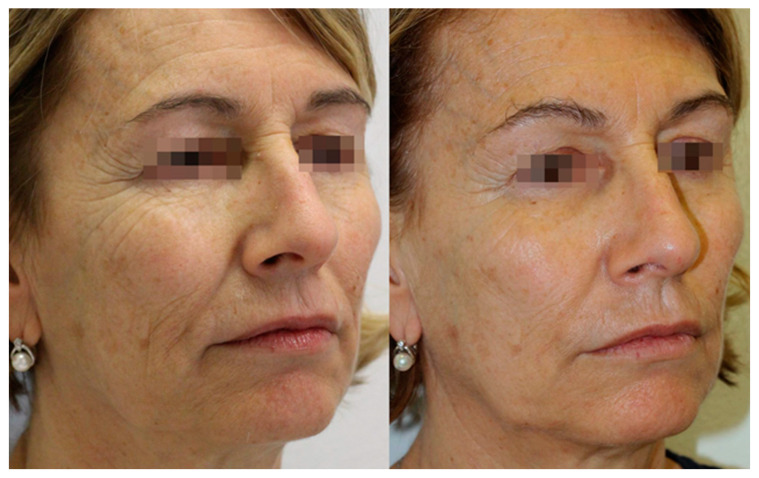
The clinical subject evaluation and photographs taken before treatment (**left side**), and three months after the beginning of the treatment (**right side**). A persistent improvement is clearly visible in the mid- (zygomatic and peri-orbital area) and lower-third (jawline and marionette lines) of the face.

**Figure 5:**
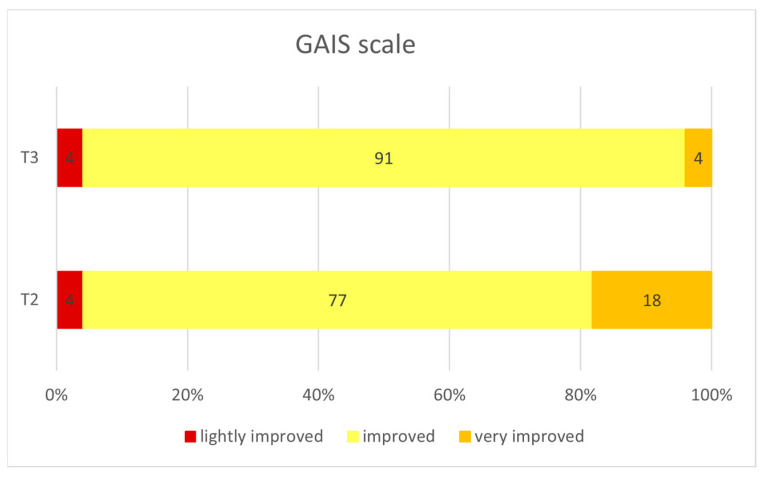
The evaluation of the patient satisfaction at T2 and T3 (thirty days and three months after the first treatment, respectively), using the Global Aesthetic Improvement Scale (GAIS). The GAIS 5-point scale ranges from a very much improved, to a worsened patient.

The instrumental evaluations were also confirmed by the patients’ self-assessment, evaluated according to the Global Aesthetic Improvement Scale (GAIS) scale (5-point scale). As shown in Figure 4, the patients showed a significant reduction in wrinkles, and a clear improvement in the general skin quality that were visible especially three months after the first treatment (T3). The amelioration was also confirmed through the GAIS evaluation (Figure 5). In this regard, 77% of subjects had already felt an improvement after the first treatment, and this positive judgement was maintained one month after the treatment (91% of subjects).

More importantly, the positive effects were obtained in the absence of any adverse reaction reported by the subjects, underlying the high tolerability of the product.

## 3. Conclusions

Profhilo Structura^®^ is a new medical device which uses the patented NAHYCO^®^ Hybrid Technology to deliver 45 mg of high- (1400 ± 200 kDa) and low- (100 ± 20 kDa) molecular-weight HA. NAHYCO^®^ Hybrid Technology has already shown a significant contribution to improving the fibroblast function and skin elasticity, both in vitro [14] and during clinical studies, with an optimal safety profile [22,23,24]. In particular, Profhilo Structura^®^ is an innovative formulation which has demonstrated a durability comparable with cross-linked HA fillers, with a higher safety profile [19]. The HCC high tolerability has also been demonstrated by a long-term safety study involving seven injections of the treatment in one year [24]. Moreover, it has also been demonstrated that Profhilo^®^ also plays pivotal roles in enhancing the viability of human ASCs, and stimulating adipogenic differentiation [18,25].

The present work is a retrospective case series, which aims to assess the bioremodeling performance of the studied medical device on the facial fat compartment of healthy volunteers, injected into the superficial fat compartment along the line from the preauricular area to the mandibular angle.

The in vitro results have demonstrated that Profhilo Structura^®^ is highly cohesive and spreadable, while it shows a lower viscosity compared to cross-linked HA dermal fillers. In this regard, the data indicate a pseudoplastic consistency that allows for easy extrusion from a syringe.

Moreover, the instrumental and clinical data taken after subjects’ treatments have demonstrated that Profhilo Structura^®^ shows high filling abilities, rendering this innovative product particularly attractive. In this context, the gel is able to optimally spread and integrate into the interstitial spaces of the adipose tissue, as demonstrated in qualitative images obtained via US scans. The US scan data on the adipose tissue restoration and repositioning after treatment with Profhilo Structura^®^ confirmed the in vitro findings on adipogenic differentiation from Stellavato et al. [18]. In particular, Stellavato et al. demonstrated that HCC plays an effective role in inducing the proliferation and differentiation of hASCs into adipocytes, compared to linear and cross-linked HA. The present article further highlights the role of HCCs and, in particular, of Profhilo Structura^®^ in adipose tissue restoration and repositioning in a clinical setting.

Profhilo Structura^®^ is the first available injectable filler with a specific indication for adipose tissue restoration, and is able to counteract facial volume loss. In this regard, the only other injectables currently available on the market able to counteract facial volume loss are represented by poly-L-lactic acid (PLLA) fillers. However, PLLA fillers are indicated for intradermal injections, and they also display a different mechanism of action to Profhilo Structura^®^, involving fibroblast recruitment and collagen production [26]. More importantly, PLLA fillers are not able to directly restore the fat tissue compartment, and they are not always free from complications, such as foreign body reactions (nodules and granulomas) [27].

Despite further evaluations being needed, in order to better characterize adipogenic markers, such as leptin, PPAR-γ, LPL, and adiponectin, after treatment with Profhilo Structura^®^, and to better investigate its long-term efficacy, this medical device could represent an effective and safer treatment for fat-tissue improvement, compared to lipofilling. Although lipotransfer is the best-known method used in fat-tissue grafting, it represents a difficult and invasive technique, with potential serious adverse events [9,10].

Taken together, our data prove Profhilo Structura^®^ as a novel and promising treatment for adipose tissue restoration and repositioning.

In particular, the quantitative analysis on skin thickness also demonstrated a general improvement in the injection area immediately after treatment and, more importantly, three months after treatment. Importantly, the quantitative results shows that the medical device not only has an immediate benefit, but that this is also maintained over time. The results have also been confirmed by clinicians and through patients’ satisfaction, as assessed by the high score evaluation on the GAIS scale.

Overall, these data demonstrate that the studied medical device shows optimal tissue integration, and has a crucial regenerating effect on the adipose tissue, leading to more relaxed skin, and a compacted area after treatment. These clinical outcomes may provide the rationale for positioning the medical device within a biorevolumetry approach, due to three simultaneous actions: an increase in the volume, a lipolifting effect, and the stimulation of adipogenic differentiation. More importantly, ProfhiloStructura^®^ allows the preservation of a natural look, and aesthetic effects similar to those obtained using cross-linked dermal fillers, avoiding any adverse effects associated with the use of different molecules from HA or other chemical molecules, and showing a high safety profile.

## 4. Materials and Methods

### 4.1. Cohesivity

The product cohesivity was evaluated by following the recent protocol reported by Sundaram et al. [20], adjusted with slight modifications. Specifically, 10 μL of toluidine blue (0.1% *w*/*w* in phosphate buffer, pH 7.4) was added to 1 g of the gel. After mixing, the samples were centrifuged (10,000× *g* 10 min) to remove air, and to obtain a homogeneous stained gel. The samples were carefully drawn into 1 mL syringes, and extruded under the reported conditions [20]. Immediately after extrusion, the magnetic stirring started, and video and images were recorded at specific time points (15, 70, and 90 s).

The cohesivity was evaluated independently by four raters that assigned, for each sample at each time point, a value of cohesivity (from 1 to 5), referring to the Gavard–Sundaram Cohesivity Scale, where 1 means fully dispersed, and 5 means fully cohesive [20]. The results were reported as the mean score ± SD.

### 4.2. Population Characteristics

This case series involved 22 subjects, aged 36–60 years, with moderate/significant mid-face volume deficit (MFVDS photographic scale: 2–3), who gave written informed consent for the use of their photographs, and for the investigation procedures, including the specific requests to maintain the same habits regarding food, exercise, make-up, cosmetics, and detergent, and to avoid undergoing ultraviolet exposure without a total sun protection cream.

The exclusion criteria were selected by referring to the medical device’s intended scope, according to the CE mark, and included pregnancy, lactation, being of a non-menopausal age and not using adequate contraception, or not being willing to perform the pregnancy test scheduled during treatment, and having a weight variation (±3 kg) during the overall treatment period. Other important contraindications to the treatment were the presence of active inflammatory skin diseases, localized or generalized infections, a known hypersensitivity/allergy to the filler components, active collagenases, hemostatic or coagulation disorders, immunological deficits, autoimmune conditions, having performed skin treatments of aesthetic correction (biomaterial implants, HA injections, radiofrequency, facelifts, Botox injections, laser treatment, or chemical peeling) in the 12 months prior to the treatment, and undergoing some pharmacological treatments (anti-inflammatory, antihistaminic, topic and systemic corticosteroids, narcotics, antidepressants, immunosuppressive drugs, and any drug able to influence the results, in the investigator’s opinion).

### 4.3. Injection Technique

The treatment consisted of the injection of 2 mL of Profhilo Structura^®^ (IBSA Farmaceutici Italia Srl, Lodi, Italy) for the hemiface using a 25 G cannula, into the superficial fat compartment along the line from the preauricular area to the mandibular angle. The injection was performed in the subcutis, at 3–4 mm depth. The protocol included a basal visit (T1), followed by the first treatment. The second treatment and visit were performed 30 days later (T2), and the follow-up visits were performed three and six months after T1 (T3 and T4, respectively).

### 4.4. Instrumental Evaluations

Esaote MyLab Five UltraSound (US) (Esaote, Italy) with a 18 Hz linear probe was used to determine the skin thickness restoration as the increase in the distance between the skin basement membrane and the parotid capsule, immediately before and after each treatment, and during the final evaluation. Moreover, the tissue integration into adipose tissue was assessed using US images. The evaluations were performed on the preauricular area of both the left and right side of the face, using transversal and parallel scans.

### 4.5. Clinical Evaluations

The evaluation of patient satisfaction with the treatment effect was determined using the Global Aesthetic Improvement Scale, a five-grade subjective test used in efficacy analysis. The investigator evaluated the aesthetic improvement in the subjects’ mid-face from their pretreatment state, according to a five-point scale: from 1 (very much improved) to 5 (a worsened patient).

The assessment of safety was based on spontaneous reporting of any adverse events or incidents by the subjects and through non leading questions by the investigators and their evaluation of local tolerability.

### 4.6. Data Analysis

The Wilcoxon signed-rank test compared the matched measurements between time points. The Mann–Whitney test was used for the differences between the measurements before and after injection. The log change was calculated using the base 2 logarithm of the ratio between the median values of the measurements of two time points. Spearman’s rank correlation assessed the relation between the measurements and time points.

## Data Availability

The data presented in this study are available on request from the corresponding author. The data are not publicly available due to privacy and ethical reasons.
